# Meal frequencies in early adolescence predict meal frequencies in late adolescence and early adulthood

**DOI:** 10.1186/1471-2458-13-445

**Published:** 2013-05-04

**Authors:** Trine Pagh Pedersen, Bjørn E Holstein, Esben Meulengracht Flachs, Mette Rasmussen

**Affiliations:** 1 National Institute of Public Health, University of Southern Denmark, Øster Farimagsgade 5, 1353 Copenhagen K, Denmark

**Keywords:** Meal frequencies, Breakfast, Lunch, Evening meal, Longitudinal study, Adolescence, Early adulthood

## Abstract

**Background:**

Health and risk behaviours tend to be maintained from adolescence into adulthood. There is little knowledge on whether meal frequencies in adolescence are maintained into adulthood. We investigated whether breakfast, lunch and evening meal frequencies in early adolescence predicted meal frequencies in late adolescence and in early adulthood. Further, the modifying effect of gender and adolescent family structure were investigated.

**Methods:**

National representative sample of 15-year-olds in Denmark with 4 and 12 year follow-up studies with measurement of breakfast, lunch and evening meal frequencies. A total of 561 persons completed questionnaires at age 15 years (baseline 1990, n=847, response rate 84.6%), age 19 years (n=729, response rate 73.2%) and age 27 years (n=614, response rate 61.6%).

**Results:**

Low meal frequencies at age 15 years was a significant predictor for having low meal frequencies at age 19 years (odds ratio (OR, 95% CI)) varying between 2.11, 1.33-3.34 and 7.48, 3.64-15.41). Also, low meal frequencies at age 19 years predicted low meal frequencies at age 27 years (OR varying between 2.26, 1.30-3.91 and 4.38, 2.36-8.13). Significant predictions over the full study period were seen for low breakfast frequency and low lunch frequency (OR varying between 1.78, 1.13-2.81 and 2.58, 1.31-5.07). Analyses stratified by gender showed the same patterns (OR varying between 1.88, 1.13-3.14 and 8.30, 2.85-24.16). However, the observed predictions were not statistical significant among men between age 15 and 27 years. Analyses stratified by adolescent family structure revealed different lunch predictions in strata.

**Conclusions:**

Having low meal frequencies in early adolescence predicted low meal frequencies in late adolescence and early adulthood. We propose that promotion of regular meals become a prioritised issue within health education.

## Background

Infrequent meal consumption has a range of adverse health consequences. Especially infrequent breakfast consumption is associated with poor nutrition intake among children, adolescents and adults [1-3] and a recent study showed that young people with irregular meal consumption had lower fruit and vegetable intake than others [4]. Breakfast skippers do not compensate for the poor nutritional intake by other meals [3]. Infrequent breakfast consumption and low frequency of meals are associated with overweight [2,3,5,6], although this observation has been challenged by a recent review [7]. Smith et al. found that low breakfast frequency in both childhood and adulthood was associated with cardio-metabolic risk factors in adulthood, e.g. higher BMI, higher mean fasting insulin, and higher LDL-cholesterol concentrations [8].

From a prevention perspective, it is important to know whether a particular risk behaviour in childhood and adolescence predicts similar behaviour in adulthood. Such prediction patterns have been described for smoking, alcohol use, physical activity, food choice, diet, and medicine use [9-14]. We have identified only two such studies about meal frequencies. Merten et al. [15] focused on the period from early adolescence to early adulthood and found that high breakfast frequency at age 14 years significantly predicted high breakfast frequency among young adults eight years later. Their analyses were controlled for several covariates such as gender, community disadvantage, and family poverty. Sweeting et al. [16] focused on the transition period from early to late adolescence and found that evening meal frequencies were maintained from age 15 until age 18 years, while fewer maintained their meal frequencies for breakfast and lunch. The analyses were stratified by gender and labour market position.

Low breakfast frequency is more prevalent among girls than boys [17-20] but little is known about gender differences in maintenance of meal frequencies over time. Sweeting et al. [16] reported that more women than men had higher breakfast frequencies and lower evening meal frequencies from age 15 to 18. Also the relevance of family structure is not well documented. The family is an important setting for the study of meals in childhood and adolescence and traditional family structure is associated with high breakfast frequency among adolescents [21-25]. It is therefore likely, although not reported in the above studies, that family structure modifies the prediction between adolescent meal frequencies and meal frequencies later in life.

Where others have used terms such as meal pattern, skipping meals, meal consumption, and regularity of meals we use the term meal frequency to characterize this behaviour.

The objective of the present study was therefore to investigate maintenance of breakfast, lunch, and evening meal frequencies over time. We used prediction analyses to study maintenance of breakfast, lunch and evening meal frequencies in two transition periods: from age 15 to 19 years, from age 19 to 27 years, and from 15 to 27 years. Further, we investigated whether these predictions were modified by gender and family structure.

## Methods

### Study design and study population

We used data from the Danish Youth Cohort, which is part of the Danish Longitudinal Health Behaviour Study (DLHBS) [9,10,26,27]. The Youth Cohort was designed to study stability and change in health behaviours over time. The study population was a national random sample of 15-year-olds, selected from the National Civic Registration System. Data about demographic factors and social background, living conditions, psychosocial factors, self-reported health, illness and symptoms, and health behaviours were collected by postal questionnaires.

The study population was 15 years old at baseline (1990) and 19 and 27 years old at the first and second follow-up study. Due to ethical concerns, the parents had the possibility to withdraw their child from the study. Of the original sample of 1100 persons, 104 parents (9.5%) did not want their child to participate, resulting in a baseline sample of 996. The response rates were 84.6% (n=843/996) at baseline, 73.2% (n=729/996) at first follow-up, and 61.6% (n=614/996) at the second follow-up. The present study included the 561 respondents (men=215, women=346) who completed the postal questionnaire in all three waves, corresponding to 56.3% of the original sample. It was not possible to compare respondents and non-respondents at baseline because the ethical review board declared that follow-up contacts to parents who declined the study to be unethical.

Ethical approval of questionnaire-based survey studies in the general population is not required in Denmark and there is no formal agency to deal with this issue. The study complies with the formal rule to request parents’ acceptance of inclusion of their children in a prospective study. The respondents were informed in writing that participation was voluntary and anonymous. The study complies with the Helsinki II declaration.

### Measurements

#### Meal frequencies

The respondents were asked how often they ate breakfast, lunch, and evening meal. These questions were almost identical in all three waves. Table 1 displays the items, response categories, and how we dichotomised and trichotomised the variables for the analyses. Breakfast and lunch items were trichotomised, but the evening meal item was dichotomised because of few observations in the category "more seldom than weekly". The term "low meal frequency" was used for the category; “less than daily”.

**Table 1:** Measurement of meal frequencies at age 15, 19 and 27 years and categorisations of variables

**Item description**	**Response categories**	**Categorisation when applied as determinant variable**	**Dichotomization when applied as outcome variable**
**Age 15**			
		**Breakfast/lunch**	
How often do you eat ....	1) Every day	1) Daily	
a) Breakfast	2) Several times a week	2) Several times a week	
b) Lunch	3) Once a week	3) More seldom	
c) Hot evening meal	4) More seldom		
		**Evening meal**	
		1) Daily	
		2) More seldom than daily	
**Age 19**			
		**Breakfast/lunch**	**All three meal types**
How often do you eat ....	1) Every day	1) Daily	1) Daily
a) Breakfast	2) Several times a week	2) Several times a week	2) More seldom than daily
b) Lunch	3) Once a week	3) More seldom	
c) Hot evening meal	4) More seldom		
	5) Never	**Evening meal**	
		1) Daily	
		2) More seldom than daily	
**Age 27**			
			**All three meal types**
How often do you eat ....	1) Every day		1) Daily
a) Breakfast	2) Several times a week		2) More seldom than daily
b) Lunch	3) Once a week		
c) Evening meal^a^	4) More seldom		
	5) Never		

^a^Observe the change in item formulation from “hot evening meal” at baseline and first follow-up to “evening meal” at second follow-up.

### Covariates

We included three covariates known to be associated with meal frequencies. Gender [17-20], adolescent family structure [21-25], and adolescent socioeconomic position measured by parents’ occupation [19,28-31].

The variable adolescent family structure was created from the 15-year-olds’ reports about who they lived with. The wording of the question was “Who do you live with?” The variable was dichotomised into traditional family structure (living with both father and mother) and other family structure (living with father or mother and/or others).

Family social class was measured by father's and mother's occupation at age 50 years, reported by the respondents at age 27. We consider this retrospective measure a measurement of childhood/adolescent social class. Occupation was coded into social class I (high) to V (low) and added social class VI for economically inactive. The coding is similar to the standards of the Danish National Institute of Social research [32] and the UK Registrar General’s classification. In order to maintain sufficient statistical power, we dichotomized social class into high (I-III) and low (IV-VI).

### Statistical analyses

In the descriptive analysis we applied chi^2^ test (assumptions for chi^2^ test were met) for gender differences and loss to follow-up and Cochrane-Armitage trend test. Analyses of prediction of meal frequencies were conducted by multivariate logistic regression analyses. Initial analyses included the full study population with adjustment for relevant covariates. Before including both family social class and family structure in the analyses we included the two variables separately due to possible correlation. We present the analyses including both covariates since the estimates did not differ significantly when including both family social class and family structure. The modifying effect of gender was examined by stratified analyses. In these analyses the determinant variables for meal consumption were dichotomised because of a limited sample size. The final step included stratification by family structure. Additionally, the modifying effects of gender and family structure were tested by inclusion of an interaction term in the statistical models analysing the full study population. Further, the full study period was studied using random effects logistic regression [33] and logistic regression with Generalized Estimating Equations (GEE) [34]. Both analyses take account of repeated measurement of observations on the same individual. Random effect models describe subject-specific estimates, and GEE describes the population averaged estimates [35]. We conducted and compared the estimates for both methods. The various analyses generated similar estimates. As we consider meal habits to be attributable to individuals, the random effect estimates are presented [35].

PROC LOGISTIC in SAS 9.2 was used to produce odds ratios for low breakfast frequency, low lunch frequency and low evening meal frequency. To avoid losing statistical power, we kept missing data on meal frequencies in the reference group to counteract for an exaggeration of statistical predictions, Table 2.

**Table 2:** Study population described by the applied variables (%, n)

	**Baseline 1990, age 15 years**			**First follow**-**up 1994, age 19 years**			**Second follow**-**up 2002, age 27 years**		
	**Total population** **(n=561) % (n)**	**Women (n=346) ** **% (n)**	**Men** **(n=215) % (n)**	**Total population (n=561) % (n)**	**Women (n=346)** **% (n)**	**Men (n=215)** **% (n)**	**Total Population** **(n=561) % (n)**	**Women (n=346)** **% (n)**	**Men (n=215)** **% (n)**
**Breakfast consumption**									
Every day	80.7 (453)	78.6 (272)	84.2 (181)	69.9 (392)	70.5 (244)	68.8 (148)	62.2 (349)^a^	67.1 (232)	54.4 (117)
Several times a week	11.4 (64)	12.1 (42)	10.2 (22)	18.4 (103)	18.8 (65)	17.7 (38)	22.3 (125)	19.4 (67)	27.0 (58)
Once a week	3.2 (18)	3.5 (12)	2.8 (6)	5.2 (29)	4.6 (16)	6.1 (13)	7.1 (40)	6.1 (21)	8.8 (19)
More seldom/never	4.1 (23)	5.2 (18)	2.3 (5)	6.2 (35)	5.5 (19)	7.4 (16)	7.1 (40)	6.9 (24)	7.4 (16)
Missing	0.5 (3)	0.6 (2)	0.5 (1)	0.4 (2)	0.6 (2)	0.0 (0)	1.3 (7)	0.6 (2)	2.3 (5)
**Lunch consumption**									
Every day	74.9 (420)	73.1 (253)	77.7 (167)	71.8 (403)	70.8 (245)	73.5 (158)	70.4 (395)	70.2 (243)	70.7 (152)
Several times a week	18.7 (105)	19.4 (67)	17.7 (38)	22.6 (127)	23.1 (80)	21.9 (47)	23.5 (132)	25.7 (89)	20.0 (43)
Once a week	1.6 (9)	1.7 (6)	1.4 (3)	2.1 (12)	2.0 (7)	2.3 (5)	2.9 (16)	1.5 (5)	5.1 (11)
More seldom/never	4.3 (24)	5.2 (18)	2.8 (6)	3.0 (17)	3.5 (11)	2.3 (5)	2.0 (11)	2.0 (7)	1.9 (4)
Missing	0.5 (3)	0.6 (2)	0.5 (1)	0.4 (2)	0.6 (2)	0.0 (0)	1.3 (7)	0.6 (2)	2.3 (5)
**Evening meal consumption**									
Every day	72.4 (406)	69.7 (241)	76.7 (165)	59.0 (331)^b^	53.5 (185)	67.9 (146)	88.6 (497)	89.0 (308)	87.9 (189)
Several times a week	26.6 (149)	29.2 (101)	22.3 (48)	33.7 (189)	37.6 (130)	27.4 (59)	10.0 (56)	10.1 (35)	9.8 (21)
Once a week	0.2 (1)	0.0 (0)	0.5 (1)	4.3 (24)	4.3 (15)	4.2 (9)	0.2 (1)	0.3 (1)	0.0 (0)
More seldom/never	0.7 (4)	1.2 (4)	0.0 (0)	2.9 (16)	4.3 (15)	0.5 (1)	0.0 (0)	0.0 (0)	0.0 (0)
Missing	0.2 (1)	0.0 (0)	0.5 (1)	0.2 (1)	0.3 (1)	0.0 (0)	1.3 (7)	0.6 (2)	2.3 (5)
**Adolescent family social class**									
Social class I							7.0 (39)	6.7 (23)	7.4 (16)
Social class II							26.0 (146)	24.0 (83)	29.3 (63)
Social class III							28.2 (158)	29.2 (101)	26.5 (57)
Social class IV							26.6 (149)	28.0 (97)	24.2 (52)
Social class V							10.9 (61)	11.0 (38)	10.7 (23)
Social class VI							0.2 (1)	0.3 (1)	0.0 (0)
Missing							1.3 (7)	0.9 (3)	1.9 (4)
**Adolescent family structure**									
Traditional	81.1 (455)	82.7 (286)	78.6 (169)						
Other	18.5 (104)	17.3 (60)	20.5 (44)						
Missing	0.4 (2)	0.0 (0)	0.9 (2)						

^a^ Gender difference p=0.003.

^b^ Gender difference p<0.001.

We conducted sensitivity analyses with other cut-points for the outcome variables breakfast and lunch frequency (for evening meal frequency it was not possible to change the cut-point). Choice of cut-points did not change the direction of the associations.

## Results

### Descriptive results

Table 2 shows that the proportions of respondents who ate breakfast daily showed a statistically significant decrease from 80.7% at age 15 years to 62.2% at age 27 years (trend test, p<0.001). The proportion of respondents who ate lunch daily was approximately 70% at age 15, 19 and 27 years. With regard to evening meal the proportion of respondents who ate an evening meal daily decreased from 72.4% at age 15 years to 59.0% at age 19 years (chi^2^, p<0.001). At age 27 years the proportion significantly increased to 88.6% (chi^2^, p<0.001).

For breakfast consumption, more women than men ate breakfast daily in the second follow-up (chi^2^, p=0.003). For lunch consumption there were no statistically significant gender differences. The proportion of men who ate an evening meal daily at baseline was larger than among women, but the difference was not statistically significant, (chi^2^, p=0.055), gender difference was significant in the first follow-up (chi^2^, p<0.001).

### Prediction analyses

#### Breakfast frequency

Table 3 shows that low breakfast frequency at age 15 years was a significant predictor of low breakfast frequency at age 19 and 27 years. Further, low breakfast frequency at age 19 years was a significant predictor for low breakfast frequency at age 27 years. We found a stronger prediction over short periods than over longer periods. When the analyses were stratified by gender we found the same patterns but among men the observed predictions were not statistically significant over the full period. No interactions with gender (age 15 to 19 years: p=0.854, age 15 to 27 years: p=0.241, age 19 to 27 years: p=0.112, Table 4) or adolescent family structure were identified (age 15 to 19 years: p=0.807, age 15 to 27 years: p=0.634, age 19 to 27 years: p=0.460, Table 5). For the full study period we found that low breakfast frequency at age 15 was a significant predictor of low breakfast frequency, Table 6.

**Table 3:** OR (CI 95%) for less than daily meal consumption at age 19 and 27 years

	**OR for breakfast/lunch/evening meal consumption less than daily at age 19**		**OR for breakfast/lunch/evening meal consumption less than daily at age 27**	
	**Unadjusted**	**Adjusted**^**a**^	**Unadjusted**	**Adjusted**^**a**^
**Age 15**				
**Breakfast consumption**				
Daily	1	1	1	1
Several times a week	**3.52(2.06-6.01)**	**3.41(1.97-5.88)**	1.64(0.97-2.79)	1.51(0.88-2.61)
More seldom	**7.11(3.56-14.22)**	**7.48(3.64-15.41)**	**2.53(1.33-4.83)**	**2.58(1.31-5.07)**
**Lunch consumption**				
Daily	1	1	1	1
Several times a week	**2.07(1.31-3.26)**	**2.11(1.33-3.34)**	**1.72(1.10-2.71)**	**1.78(1.13-2.81)**
More seldom	**4.56(2.21-9.43)**	**4.34(2.07-9.10)**	1.67(0.79-3.50)	1.76(0.83-3.73)
**Evening meal consumption**				
Daily	1	1	1	1
More seldom than daily	**3.69(2.50-5.44)**	**3.39(2.28-5.04)**	1.37(0.76-2.45)	1.23(0.67-2.27)
**Age 19**				
**Breakfast consumption**				
Daily			1	1
Several times a week			**2.71(1.74-4.23)**	**2.74(1.74-4.31)**
More seldom			**2.52(1.48-4.31)**	**2.26(1.30-3.91)**
**Lunch consumption**				
Daily			1	1
Several times a week			**2.77(1.82-4.22)**	**2.81(1.84-4.28)**
More seldom			1.79(0.80-3.99)	1.42(0.60-3.35)
**Evening meal consumption**				
Daily			1	1
More seldom than daily			**4.31(2.35-7.89)**	**4.38(2.36-8.13)**

^a^Adjusted for gender, adolescent family social class and adolescent family structure.

**Table 4:** Adjusted OR (CI 95%) for less than daily meal consumption at age 19 and 27 years, stratified by gender^a^

	**OR for breakfast/lunch/evening meal consumption less than daily at age 19**			**OR for breakfast/lunch/evening meal consumption less than daily at age 27**		
	**Women**	**Men**	**P-value gender interaction**^**b**^	**Women**	**Men**	**P-value gender interaction**^**b**^
**Age 15**						
**Breakfast consumption**						
Daily	1	1		1	1	
More seldom than daily	**4.57(2.60-8.03)**	**4.73(2.13-10.52)**	0.854	**2.25(1.31-3.89)**	1.27(0.59-2.73)	0.241
**Lunch consumption**						
Daily	1	1		1	1	
More seldom than daily	**2.68(1.61-4.48)**	**2.20(1.09-4.43)**	0.638	**1.88(1.13-3.14)**	1.55(0.75-3.20)	0.481
**Evening meal consumption**						
Daily	1	1		1	1	
More seldom than daily	**2.80(1.72-4.55)**	**5.64(2.79-11.42)**	0.132	1.07(0.50-2.27)	1.64(0.58-4.62)	0.544
**Age 19**						
**Breakfast consumption**						
Daily				1	1	
More seldom than daily				**1.98(1.21-3.23)**	**3.77(2.02-7.03)**	0.112
**Lunch consumption**						
Daily				1	1	
More seldom than daily				**1.98(1.20-3.26)**	**4.00(2.02-7.93)**	0.140
**Evening meal consumption**						
Daily				1	1	
More seldom than daily				**2.90(1.36-6.18)**	**8.30(2.85-24.16)**	0.132

^a^Adjusted for adolescent family social class and adolescent family structure.

^b^The interaction term was included in the analyses of the full study population.

**Table 5:** OR (CI 95%) for less than daily meal consumption at age 19 and 27 years, stratified by adolescent family structure^a^

	**OR for breakfast/lunch/evening meal consumption less than daily at age 19**			**OR for breakfast/lunch/evening meal consumption less than daily at age 27**		
	**Traditional family structure**	**Other family structure**	**P-value adolescent family structure interaction**^**b**^	**Traditional family structure**	**Other family structure**	**P-value adolescent** ** family structure interaction**^**b**^
**Age 15**						
**Breakfast consumption**						
Daily	1	1		1	1	
More seldom than daily	**4.63(2.73-7.84)**	**4.34(1.72-10.97)**	0.807	**1.76(1.05-2.95)**	2.21(0.89-5.52)	0.634
**Lunch consumption**						
Daily	1	1		1	1	
More seldom than daily	**2.50(1.56-3.99)**	**2.49(1.03-6.03)**	0.935	**2.19(1.37-3.48)**	0.72(0.27-1.90)	0.080
**Evening meal consumption**						
Daily	1	1		1	1	
More seldom than daily	**3.41(2.20-5.29)**	**4.21(1.55-11.41)**	0.909	1.09(0.60-2.15)	2.74(0.61-12.38)	0.305
**Age 19**						
**Breakfast consumption**						
Daily				1	1	
More seldom than daily				**2.38(1.55-3.64)**	**3.91(1.58-9.68)**	0.460
**Lunch consumption**						
Daily				1	1	
More seldom than daily				**3.11(1.99-4.84)**	0.96(0.37-2.55)	**0**.**038**
**Evening meal consumption**						
Daily				1	1	
More seldom than daily				**3.71(1.95-7.06)**	**Not statistical possible because of too few cases**	0.951

^a^Adjusted for gender and adolescent family social class.

^b^The interaction term was included in the analyses of the full study population.

**Table 6:** OR (CI 95%) for less than daily meal consumption at age 19 and 27 years, random effect model

	**OR for breakfast consumption less than daily at age 19 and 27**	**OR for lunch consumption less than daily at age 19 and 27**	**OR for evening meal consumption less than daily at age 19 and 27**
	**Adjusted**^**a**^	**Adjusted**^**a**^	**Adjusted**^a^
**Breakfast/lunch/evening meal consumption, age 15**			
Daily	1	1	1
Several times a week	**2.28(1.49-3.50)**	**1.95(1.57-5.03)**	..^**b**^
More seldom	**4.44(2.58-7.64)**	**2.81(1.57-5.03)**	**2.49(1.75-3.54)**

^a^Adjusted for age, gender, adolescent family social class and adolescent family structure.

^b^Evening meal item was dichotomised because of few observations in the category "more seldom".

#### Lunch frequency

Table 3 shows that low lunch frequency at age 15 years was a strong predictor of low lunch frequency at age 19 years. Low lunch frequency at age 15 and age 19 years predicted low lunch frequency in early adulthood. As for breakfast we found a stronger prediction over short periods than over longer periods. Stratified by gender the same patterns occur but among men the observed predictions were not statistically significant over the full period Testing for gender interaction showed no statistically significant differences (age 15 to 19 years: p=0.638, age 15 to 27 years: p=0.481, age 19 to 27 years: p=0.140, Table 4). Stratification by adolescent family structure suggested that family structure was an effect modifier in the prediction between low lunch frequency in adolescence and low lunch frequency in early adulthood. Among the persons living in a traditional adolescent family structure the odds ratio was high varying between 2.19 and 3.11 and among the persons living in other family structures the odds changed direction, OR varying between 0.72 and 0.96, Table 4. Statistical testing for adolescent family structure interaction supported the observed modifying effect (age 15 to 19 years: p=0.935, age 15 to 27 years: p=0.080, age 19 to 27 years: p=0.038, Table 5). Studying the full study period we found that low lunch frequency at age 15 was a significant predictor of low lunch frequency, Table 6.

#### Evening meal frequency

Low evening meal frequency at age 15 years was a significant predictor for low evening meal frequency at age 19 years, but we found no prediction from age 15 to 27 years. Further, we found an increased prediction for having low evening meal frequency at age 27 years if the respondent had low evening meal frequency at age 19 years. As for breakfast and lunch we found a stronger prediction over short periods than over longer periods. Analyses stratified by gender showed that low evening meal frequency at age 15 years among both women and men was an predictor for having low evening meal frequency at age 19 years, but the probability was stronger among men (women OR=2.80, 1.72-4.55, men OR=5.64, 2.79-11.42). The same gender differences was found from age 19 to 27 years (women OR=2.90, 1.36-6.18, men OR=8.30, 2.85-24.16). However, no statistical interaction with gender was identified (age 15 to 19 years: p=0.132, age 15 to 27 years: p=0.544, age 19 to 27 years: p=0.132, Table 4). Stratification by adolescent family structure showed no modifying effect of adolescent family structure (age 15 to 19 years: p=0.909, age 15 to 27 years: p=0.305, age 19 to 27 years: p=0.951, Table 5). Studying the full study period we found that low evening meal frequency at age 15 was a significant predictor of low evening meal frequency, Table 6.

### Loss to follow-up

We examined differences in baseline characteristics among respondents with and without data at the first follow-up and at the second follow-up, and between first follow-up and second follow-up. Significantly more men than women were in the loss to follow-up group in all three analyses (p=0.028, p<0.001, p<0.001). We found a borderline statistically significantly larger proportion from the loss to follow-up group in low family social class (p=0.051). With regard to breakfast and lunch we found that a significantly larger proportion ate breakfast (p=0.002) and lunch (p=0.016) less than daily at age 15 year among the loss to follow-up group. Further, we conducted the prediction analyses including all missing observations as worst case response and afterwards as best case response in the meal frequency and covariate variables. This did not change the magnitude or direction of the estimates.

## Discussion

This is the first longitudinal study which examined prediction of breakfast, lunch and evening meal frequencies over two important transition periods in young people’s life from age 15 to 19, 19 to 27 and 15 to 27 years.

The proportion of respondents who ate breakfast daily decreased continuously from adolescence into early adulthood. The proportion who ate lunch daily was constant over the period, while the proportion who ate an evening meal daily decreased from early adolescence into late adolescence and then increased into early adulthood. Lytle et al. [36] studied cross-sectional prevalence for all three meal types among third, fifth and eighth graders. The study population was younger than ours but the patterns were the same. Sweeting et al. [16] found that the prevalence of breakfast and lunch decreased with age and the prevalence of the evening meal remained constant over the period. Niemeier et al. [37] found that breakfast consumption decreased with age from adolescence into early adulthood. We hypothesize that the decrease in eating breakfast with increasing age is linked to the transition from living at home with the family of origin and moving to live alone. In the family, breakfast may be more available and it may be more appealing to eat breakfast living in a family setting. Regarding the findings for the evening meal, some of the changes observed in prevalence may ascribe to the change from hot evening meal to evening meal in the item formulation. However, it could be hypothesized that people tend to have higher evening meal frequency in adulthood due to family obligations.

We found that low meal frequencies in early adolescence predicted low meal frequencies in late adolescence. Further, low breakfast and lunch frequencies in early adolescence predicted low breakfast and lunch frequencies in early adulthood. Low meal frequencies in late adolescence predicted low meal frequencies in early adulthood. The predictions were stronger over shorter than longer periods. For breakfast frequency, Merten et al. [15] found that high breakfast frequency in adolescence predicted high breakfast frequency in early adulthood. Their study period was shorter than that of our study, but the findings are consistent. Sweeting et al. [16] studied the stability in meal frequencies from adolescence into late adolescence. Therefore, it is not possible to compare our results directly with their findings. Their findings showed that evening meal frequencies was constant over the period, while breakfast and lunch frequencies decreased. To some extent, their findings are consistent with our observations.

The presented results of the modifying effect of gender showed that among women, low meal frequencies in early adolescence predicted low meal frequencies in early adulthood, but we did not find similar predictions among men. Further, we found a stronger prediction among men than among women for evening meal frequency from adolescence into late adolescence and from late adolescence into early adulthood. In the study by Sweeting et al. [16] the changes in meal frequencies are stratified by gender. They found that men to a higher degree than women maintained their evening meal frequency from adolescence into late adolescence. It seems that the predictions, especially among men, are stronger over shorter periods than over longer periods.

We found that family structure modified the prediction between low lunch frequency in early/late adolescence and low lunch frequency in early adulthood. Our findings indicate that living in a non-traditional family structure in adolescence decreases the prediction of low lunch frequency. Nevertheless, no other studies have investigated whether adolescent family structure influences prediction of meal frequencies over time. Cross sectional studies show that living in two-parent families is positively associated with high meal frequency in adolescence [21,23,28,38-41]. This is not consistent with our longitudinal findings. If the measurement of adolescent family structure had been more refined, we would have been able to differentiate the category of other family structures, which covers a broad range of family constellations.

The strengths of this study are high response rates, a national representative sample and a long follow-up period that includes two important transition periods. Further, the prediction analyses have been conducted using both by simple logistic regressions, GEE-models and random effect logistic regression models. The findings are consistent between models. The findings must also be seen in relation to the limitations of the study. There is a risk of selection bias because the original sample was reduced by the 11% of parents who declined to allow their child to participate in the study. We are unable to provide further information to substantiate this issue. The loss to follow-up may also cause selection bias. We found significantly higher loss to follow-up among respondents with the following characteristics: men, low adolescent family social class, and low breakfast and lunch frequency. This may have influenced the generated results. However, worst and best case missing analyses showed no discrepancy from the generated results. Information bias may exist in our study due to using self-reported data. Also the slight change in item formulation for the evening meal for the second follow-up could introduce information bias. However, it is unlikely that this change compromised the observed prediction patterns.

Studies about meal consumption suffer from difficulties in defining a meal. Denmark has a Nordic meal culture of three main meals a day [42], and people use the concepts breakfast, lunch and evening meal in their daily life. Nevertheless, it is not clear what constitutes a proper meal and how the respondents interpret the term. The literature is limited regarding the use of frequency questionnaires for measuring meal consumption. A frequency question on breakfast consumption was validated in the Health Behavior in School-Aged Children (HBSC) study in 2004–2005. The validation showed a moderate accordance with food habits diaries (kappa statistics 0.47) [43]. The formulation of the question was similar to the formulation in this study but not identical. More validation studies of frequency questionnaires for measuring meal consumption are needed.

In every study possible unmeasured confounders must be considered. Other studies have found ethnic differences in diet and breakfast consumption [44,45] and it would have had been relevant to include ethnic background as a confounder. However, our dataset does not provide information on ethnic background. Several studies have found an association between meal frequencies and BMI [2,3,5,6], but we abstained from including BMI as a covariate because it is unclear whether BMI confound or mediate the association. Further, aspects of family life such as parents’ working hours, family coherence and child-raring styles may also confound the findings but we didn’t have data to investigate these ideas.

## Conclusion and implications

Our study contributes to the existing literature by showing that low breakfast, lunch and evening meal frequencies strongly predicts low meal frequencies from early and late adolescence into early adulthood and moderately from late adolescence into early adulthood. The patterns of predictions were persistent over a long follow-up period that includes two important transitions. In future studies a conceptual clarity is needed e.g. there is a lack of a precise meaning of terms like meal skipping, frequency and regularity. More detailed measurements, for example, separate analyses for meals on weekdays and meals during weekends would provide more refined analyses. Additionally, we propose studies that will provide deeper insight into which factors contribute to the considerable changes in meal frequencies from early adolescence and onwards.

The practical implications of this study suggest that it is essential to promote regular meal consumption in early and late adolescence. In promoting regular meal consumption it is relevant to include relevant settings for adolescents’ meal consumption such as the family and school.

## Competing interests

The authors declare that they have no competing interests.

## Authors’ contributions

TPP, BEH and MR contributed to planning of research questions and analytical strategy. Data analyses were conducted by TPP. TPP drafted the manuscript with critical input from BEH, EMF and MR. All authors have read and approved the final manuscript.

## Pre-publication history

The pre-publication history for this paper can be accessed here:

http://www.biomedcentral.com/1471-2458/13/445/prepub
